# Metabolomic Analysis Revealed Distinct Physiological Responses of Leaves and Roots to Huanglongbing in a Citrus Rootstock

**DOI:** 10.3390/ijms23169242

**Published:** 2022-08-17

**Authors:** Qing Chen, Ailing Min, Shu Luo, Jinwei He, Runqin Wu, Ximeng Lin, Yan Wang, Wen He, Yunting Zhang, Yuanxiu Lin, Mengyao Li, Yong Zhang, Ya Luo, Haoru Tang, Xiaorong Wang

**Affiliations:** 1 College of Horticulture, Sichuan Agricultural University, Chengdu 611130, China; 2 Institute of Pomology and Olericulture, Sichuan Agricultural Universtiy, Chengdu 611130, China

**Keywords:** Huanglongbing, *Citrus junos*, metabolomic alterations

## Abstract

Huanglongbing (HLB) is an obstinate disease in the citrus industry. No resistant citrus resources were currently available, but various degrees of Huanglongbing tolerance exist in different germplasm. *Citrus junos* is emerging as one of the popular rootstocks widely used in the citrus production. However, its responses to the HLB causal agent, *Candidatus* Liberibacter asiaticus (CLas), were still elusive. In the current study, we investigated the physiological, anatomical, and metabolomic responses of a *C. junos* rootstock ‘Pujiang Xiangcheng’ by a controlled CLas grafting inoculation. The summer flushes and roots were impaired at 15 weeks after inoculation, although typical leaf symptomatic phenotypes were not obvious. The chlorophyll pigments and the photosynthetic rate were compromised. The phloem sieve tubes were still working, despite the fact that the callose was deposited and the starch granules were accumulated in the phloem cells. A wide, targeted metabolomic analysis was carried out to explore the systematic alterations of the metabolites at this early stage of infection in the leaves and root system. The differentially accumulated metabolites in the CLas-affected leaves and roots compared with the mock-inoculation control tissues revealed that distinct responses were obvious. Besides the commonly observed alteration of sugar and amino acids, the active break down of starch in the roots was discovered. The different types of fatty acids were altered in the two tissues, with a more pronounced content decline in the roots. Our results not only provided fundamental knowledge about the response of the *C. junos* rootstock to the HLB disease, but also presented new insights into the host–pathogen interaction in the early stages.

## 1. Introduction

Huanglongbing (HLB), also known as citrus greening, is an obstinate disease in the citrus industry. Since its first discovery a century ago, HLB has been transmitted throughout the main production area all over the world. It causes tremendous economic losses for the citrus production and processing industry. In Florida, it has caused 73% loss of citrus production (USDA, 2021). In China, HLB incidences were reported in more than 300 counties of 10 provinces, with over 50 million citrus trees being destroyed [1,2]. Despite the tremendous progress in understanding the transmission and the plant responses to the disease, we still encountered great challenges in understanding the pathogenesis of the disease, partially ascribed to the hindrance that the causal agent *Candidatus* Liberibacter spp. is yet to be cultured in artificial media. *Ca*. L. asiaticus (CLas), *Ca*. L. americanus (CLam), and *Ca*. L. africanus (CLaf) were the main causal agents found in the HLB-endemic regions, with the CLas discovered to be the most prevalent and destructive one [3,4]. They are transmitted by the pest vector, citrus psyllid (*Diaphorina citri*), and through agricultural practices, such as grafting using contaminated budwood. Consequently, the control of the pest vector, early discrimination of the infected plants, and mitigation of the growth impairment were the main practices in dealing with the disease.

The HLB disease has brought systematic growth disorders to the trees. The asymmetric blotchy mottle in the leaves and yellow shoots are the typical visual signatures for the HLB occurrence. The asymmetrical pattern helps to distinguish it from the symptoms of nutrient deficiencies [5]. The mottled leaves usually come in several months or even years after the bacteria reach a certain criterion, depending on the tolerance of the citrus plants and the environmental conditions [6]. The diseased fruits stay a green color at the stylus end during the ripening stage, with a distorted or lopsided shape and ruined quality [7,8]. The root system is destroyed before the aboveground visible symptoms [9,10,11]. This root decay is always accompanied with even and consistent CLas accumulations [10,12]. A premature fruit drop and irregular leaf loss can happen. All of these factors lead to the sudden drop in fruit yield, before the tree eventually dies. The anatomical observations reveal that these disorders are associated with the phloem callus deposition, phloem cell wall, and cambium thickening [12,13]. The dysfunction of the sieve tubes and companion cells is observed in the severely infected materials [14].

Besides these visible alterations, extensive systemic physiological and molecular changes were also reported as being uncovered through transcriptomic, proteomic, or metabolomic approaches. In particular, the gene expression and proteomic profile comparison between HLB-tolerant and sensitive varieties revealed several candidate targets of CLas, and have inferred the possible pathways that the host plant utilizes to fight the bacteria invasion [11,12,15,16,17,18,19,20,21,22,23,24,25]. The components involved in the defense system, cell wall integrity, energy production, and catabolism were among the most disturbed pathways. To overcome the shortcomings of the transcriptome and proteome assays, metabolomic profiling was also used to dissect the physiological changes of the plant’s response to HLB. The accumulations of sugars, amino acids, and fatty acids, as well as several secondary metabolites, such as flavonoids, were significantly remodeled [26,27,28,29,30]. However, the trends varied from species to species, and tissues to tissues.

The recent investigations demonstrated that the root system was the obligatory colonization place of CLas in the early stages [9,10,30]. The root acts as a reservoir tissue for CLas replication and a redistribution source for new infections in other tissues. The decreased water and mineral nutrient uptake in the root further exacerbate the effects of HLB [31]. Callus deposition and starch accumulation were not observed in the roots, in contrast to that in the leave tissues of CLas-infected trees [10]. Disclosing the plants’ responses in the roots might be as important as those in the leaves for understanding the plant–pathogen interactions, which can provide valuable information for developing new strategies in controlling the disease. Despite the advances in HLB research in the leaf and fruit tissues, the systematic physiological responses to the CLas in the root are still to be thoroughly investigated. Even less is known about the synergetic or specific response in the root and canopy. *Citrus junos* is emerging as one popular rootstock widely used in the citrus production [32]. However, its susceptibility/tolerance to HLB was still elusive. In this study, we were aiming to: (1) investigate the overall responses of this new citrus rootstock to the CLas pathogen attack from the aspect of physiological and anatomic changes; and (2) explore and compare the metabolomic alterations in the leaves and roots at the early asymptomatic stage. Our results uncovered obviously distinct physiological responses in the roots and leaves, which provided new insight into the plant–pathogen interactions among the different tissues.

## 2. Results

### 2.1. Detection of CLas in the Leaves Using Quantitative PCR

To confirm that the HLB bacteria were amplified and colonized in the leaf tissues, RT-qPCR was performed 15 weeks after graft-inoculation (wai). The pathogenic bacteria were detected in all of the grafted ‘Pujiang Xiangcheng’ materials (infection rate, 100%) but not in the mock-inoculation trees. The cycle number of RT-qPCR ranged from 23.75 to 24.60. The corresponding absolute number of CLas was 3.96 to 7.66 × 10^5^ copies/μg DNA, indicating that a large amount of CLas were accumulated in the leaves’ midveins.

### 2.2. Growth Impairment of Pujiang Xiangcheng Due to CLas Infection

**Figure 1:**
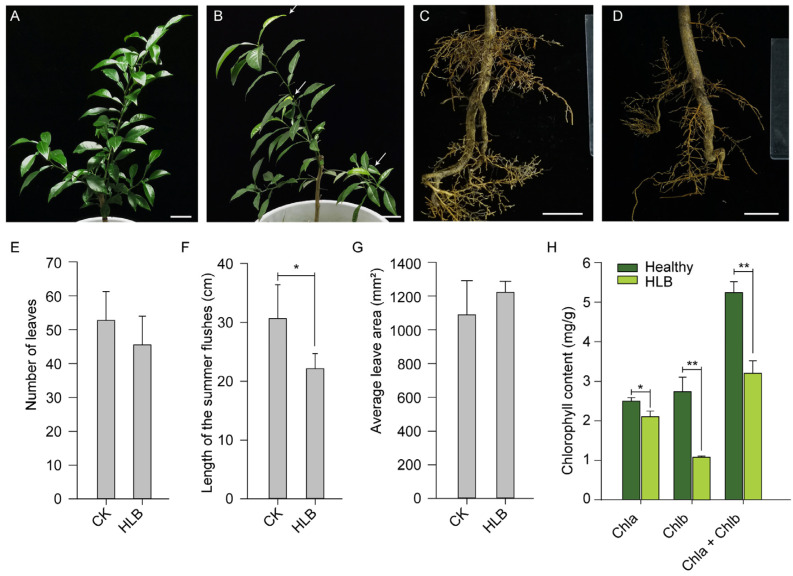
Growth impairment of Pujiang Xiangcheng due to CLas infection 15 weeks after inoculation. (**A**): The phenotypic appearance of the leaves and flushes in the mock-inoculated plants; (**B**): Growth status of the above ground part of *C. junos* positive for CLas; (**C**): The new growth of fibrous roots in the control plants; (**D**): The ruined root system in the HLB trees. The number of leaves (**E**); length of the summer flushes (**F**); average leaf area of the mature leaves (**G**); and the chlorophyll content of the leaves (**H**) were compared. Statistical analysis was completed by using a Student’s *t* test, significances were labeled as * (*p* < 0.05) and ** (*p* < 0.01). The white scale bar in (**A**–**D**) is 5 cm. The bar plots in (**E**–**H**) were presented as mean ± standard deviation (*n* = 3).

Three months after the graft inoculation with CLas, no typical blotchy mottled symptoms were observed. However, a few young leaves started to manifest chlorosis, and the growth vigor of the infected plants was slightly weaker than that of the control plants (Figure 1). The most significant differences were observed for the summer flush growth, as well as in the root growth (Figure 1B,D). The number and the area of the mature leaves were almost the same between the two groups. A few terminal young leaves had a slight yellowish color (white arrows in Figure 1B), while the control plants had dark green color (Figure 1A). In line with these results, the chlorophyll content of the leaves was significantly lower in the infected group (Figure 1H). Moreover, strikingly fewer fibrous roots were observed in the CLas-infected trees (Figure 1C,D) although the root mass was not quantified. When sampling the root system, we found that the roots of the diseased plants were partially necrotic and black. The epidermis of the damaged root systems was easily separated from the inner layer of the roots. These results indicated that the roots had been destroyed by the pathogen, and the leaf symptoms were beginning to develop.

### 2.3. Photosynthetic Changes

**Figure 2:**
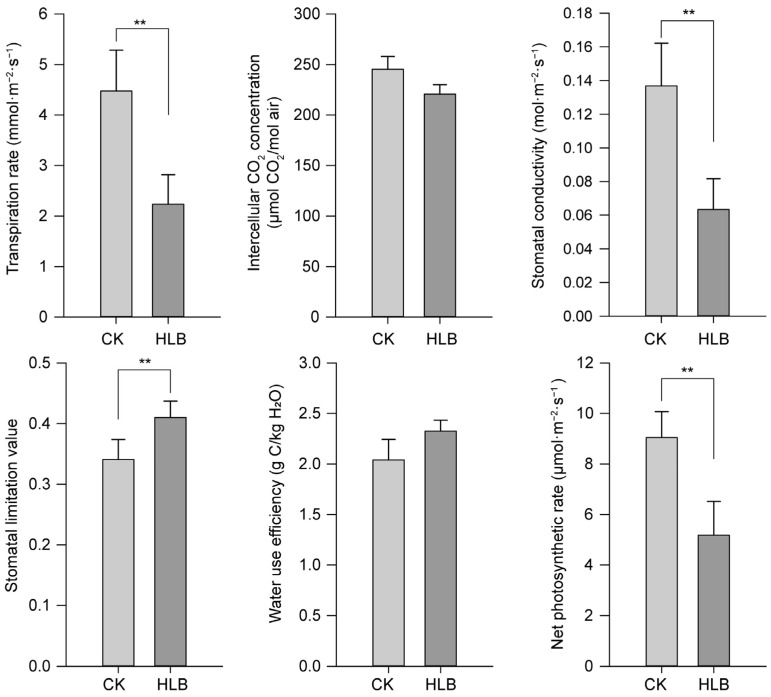
Changes of photosynthetic parameters in leaves of ‘Pujiang Xiangcheng’ after HLB infection. ** indicates extremely significant difference (*p* < 0.01) using a Student’s *t*-test. The bar plots were presented as mean ± standard deviation (*n* = 3).

The photosynthetic parameters were tested in the infected leaves as well as the control plants using a portable Li-6800XT, 90 days after the pathogen inoculation. The results showed that the tested photosynthesis-related parameters had significantly decreased in the HLB plants (Figure 2), except the leaf-water utilization rate and the intercellular carbon dioxide concentration. The net photosynthetic rate decreased from 9.04 µmol m^−2^ s^−1^ to 5.18 µmol m^−2^ s^−1^. The leaf transpiration rate decreased to almost half of the control plants. Similar trends were observed for the stomatal conductance. In correspondence, the stomatal limitation values in the leaves of the infected plants were significantly higher than those of the control plants, indicating that an insufficient CO_2_ supply due to changes in the stomatal conductance and the reduced leaf transpiration rates were the main limiting factors for their photosynthesis.

### 2.4. Changes of the Antioxidant Enzyme Activity in the Leaves

In the previous reports, the CLas infection caused a burst of reactive oxygen species in the CLas-positive flush, which in turn could influence the activity of the antioxidant enzyme composition and activity [33]. The activity of the main antioxidant enzymes including the superoxide dismutase (SOD), peroxidase (POD), and catalase (CAT) were tested in the leaf tissues of ‘Pujiang Xiangcheng’. The extremely significant decrease, and significant decreases in the CAT and SOD activity were observed, respectively (Table S1, Supplementary Materials). In contrast, the activity of POD did not fluctuate too much.

### 2.5. Anatomy Modifications of the Citrus Midribs Induced by HLB

**Figure 3:**
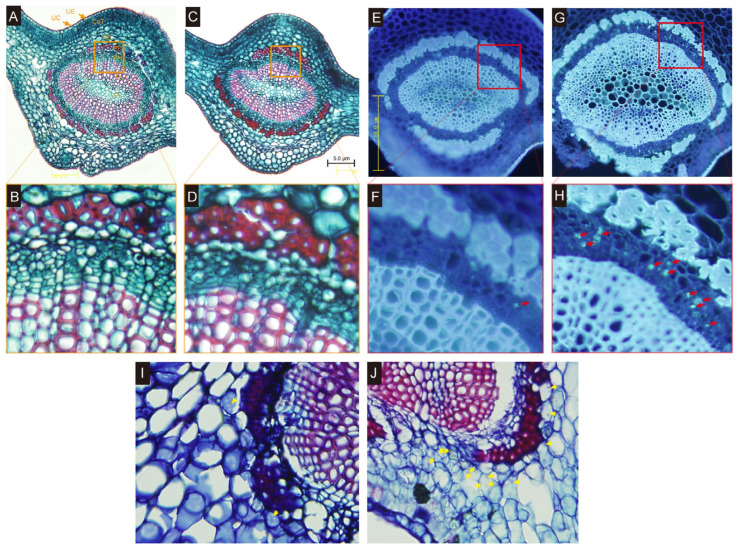
Anatomy modifications of the citrus midribs induced by HLB. Safranin O staining results of the phloem fibers in the control (**A**,**B**) and HLB plants (**C**,**D**). (**B**,**D**) were magnified graph of the orange rectangle-frame in (**A**,**C**), respectively. Aniline blue callose deposition in the phloem of CLas-infected plants (**G**,**H**) and healthy plants (**E**,**F**). The fluorescence of the stained callose was marked using red arrows. (**F**,**H**) were the magnified red rectangle graph-frame in (**E**,**G**), respectively. The accumulation of starch grains stained by methylene blue-azure II-basic fuchsin in the phloem and parenchyma cells of HLB leaves (**J**) and mocked control plants (**I**). The stained starch grains were marked using yellow arrows. UC: upper cuticle; UE: upper epidermis; CoT: collenchyma tissue; PC: parenchyma cell; PF: phloem fiber; Ph: phloem; Xy: xylem; Pi: pith.

The midribs of the HLB leaves (three months after inoculation(3 mai)) were subjected to anatomic observation in comparison with the healthy leaves. The safranin O staining results demonstrated that the phloem fibers were dramatically thickened in the CLas-infected leaves (dark red color, Figure 3C,D) compared to the control samples (Figure 3A,B). The number of fiber cell layers was also increased, relative to the healthy leaves. Moreover, the infected leaf tissues suffered severe cell necrosis, characterized by misshapen or collapsed phloem cells (Figure 3D). The cell boundaries were not as clear as those of the non-infected leaves. A large amount of callose was deposited in the sieve pores of the phloem in the HLB materials, which began to plug the phloem (Figure 3G,H). However, there were still large proportions of the sieve pores that were not completely blocked, indicating that the normal nutrient-transporting could still function in the phloem. In contrast, the callose accumulation was occasionally seen in the control materials (Figure 3E,F). Through basic fuchsin-methylene blue staining, a large accumulation of starch granules could be clearly observed in the phloem and parenchyma cells of the leaf veins of the HLB-infected material (Figure 3J). In contrast, a few cells in the control material showed an accumulation of starch granules (Figure 3I).

### 2.6. Metabolomic Changes in the Leaves and Roots

**Figure 4:**
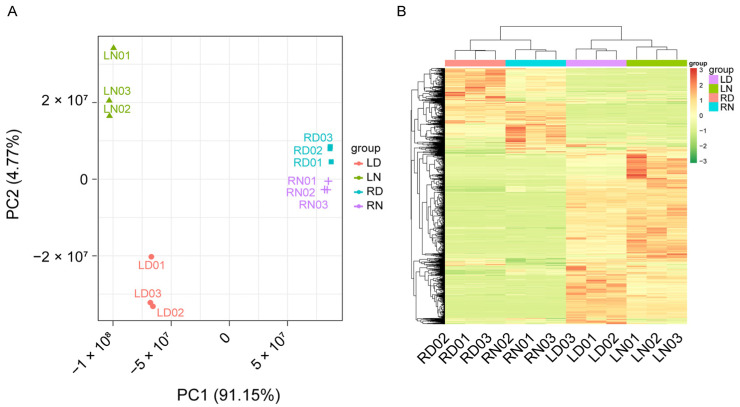
Principal component analysis (**A**) and hierarchical clustering results (**B**) using all detected metabolite changes in ‘Pujiang Xiangcheng’ leaves and roots with HLB infection. LD: leaf with HLB disease; LN: healthy leaf tissue; RD: root with HLB disease; RN: normal healthy root tissue.

To further clarify the physiological changes of ‘Pujiang Xiangcheng’ infected with the Huanglongbing pathogen, we measured the changes in the various metabolites in the leaves and roots of the infected but asymptomatic materials, with reference to the negative-mock control plants. A total of 1253 different compounds were detected, covering a broad spectrum of both primary and secondary metabolites (Table S2, Supplementary Materials). The principal component analysis (PCA) and clustering (Figure 4) showed that the variation between the replicates within a group was much smaller than the between-group variation. The PCA also classified the samples into the root or leaf tissues, and the healthy or diseased tissues on the principal component 1 (PC1) and PC2 axis. These two components explained 91.15% and 4.77% of the total variation, respectively (Figure 4A). The results also demonstrated that the stronger metabolomic changes had occurred in the leaves than in the roots, due to the impact of CLas infection. The clustering results further confirmed the results of the principal component analysis (Figure 4B).

A discriminant model was firstly optimized to maximize the discriminating ability between the healthy and the infected status in the leaves or in the roots by using the partial least squares-discriminant analysis (PLS-DA) and its extension, orthogonal partial least squares-discriminant analysis (OPLS-DA) method. A seven-fold cross validation (CV) was employed to obtain the R^2^ and Q^2^ values, which reflect the predictive ability and the explained variance of the built model. Based on the results, the healthy status was discriminated with an R^2^X of 0.86, an R^2^Y of 1.0, and a Q^2^Y of 0.977 in the leaves (Figure S1A, Supplementary Materials). Likewise, the model constructed by the root metabolites was equally valid for distinguishing the healthy and HLB status (Figure S1B, Supplementary Materials). Based on the obtained discrimination model, the metabolites with a variable importance in the projection (VIP) value greater than one were selected for further analysis.

The relative fold change (FC) of each metabolite was directly computed between the two comparison groups. Further, the *p* value was calculated via the heteroscedastic *t*-tests with the two-tail distribution. Using the pre-defined criteria (VIP > 1, |FC| > 1, and *p* < 0.05), a total of 722 differentially accumulated metabolites (DAMs) were detected as the most important variables for differentiating HLB and CK in the leaves and roots. Among them, 404 DAMs were detected in the infected/healthy leaf group, of which 231 were upregulated, and 173 were downregulated. Significant differential accumulations of 318 compounds were detected in the roots group, among which 178 were upregulated, while 140 were reduced. A detailed classification of these compounds demonstrated that the HLB pathogen had provoked significant changes in both the plants’ primary and secondary metabolism.

**Figure 5:**
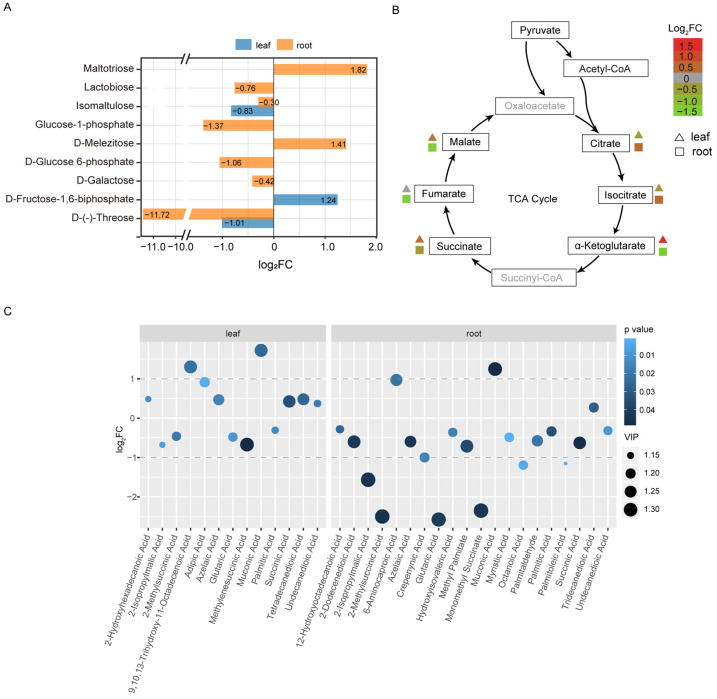
The alteration of sugars, intermediate products in the tricarboxylic acid (TCA) cycle, and fatty acids content in the CLas-infected *C. junos* leaves and roots. (**A**): Differentially accumulated sugars; (**B**): Opposite change trends of the products of the TCA cycle. The undetected intermediates were gray colored and the detected ones in this study were in black font color. (**C**): Bubble plot indicating the alterations of the differentially regulated fatty acids. Only the compounds with a VIP value >1, change extent with *p* < 0.05 and fold change >1 were included.

The D-(−)-threose and isomaltulose were depleted in both the root and leave tissues (Figure 5A). Particularly, threose was more than 1000 times less than that of the healthy tissues. The intermediates of the biosynthesis of starch, including the glucose 1-phosphate and the glucose 6-phosphate in the root library, were significantly decreased. In contrast, the starch debranching indicator, maltotrioses, were dramatically augmented (Figure 5A). The D-galactose and lactobiose were reduced in the roots, while D-melezitose was accumulated. In the HLB leaves, fructose-1,6-biphosphate was increased to 2.37 times higher than that in the healthy leaves. We further investigated the changes in the products involving the tricarboxylic acid (TCA) cycle. Interestingly, of the detected metabolites, most showed the opposite accumulation trends in the roots and leaves (Figure 5B).

Among all of the detected metabolites, 20 amino acids were included. In the leaves, four amino acids were differentially accumulated, ascribed to HLB. Leucine and isoleucine were significantly upregulated, but glutamic acid and methionine were significantly downregulated. In the root system, seven amino acids were differentially accumulated. The content of cysteine, leucine, isoleucine, and phenylalanine increased significantly, with the most prominent increase occurring in the cysteine, reaching more than four times that in the healthy roots. In contrast, the glutamic acid, aspartic acid, and the methionine content significantly declined.

Forty-two fatty acids and conjugates were detected in this assay (Table S3, Supplementary Materials). Twenty-five of the molecules were differentially accumulated, with 13 in the leaves, and 20 in the root tissues (Figure 5C). In the leaves, eight fatty acids and derivatives (61.5%) were upregulated. Muconic acid and 9,10,13-trihydroxy-11-octadecenoic acid were mostly altered by the diseases (>2-fold change). In the roots, the majority of the detected fatty acids were downregulated (17/20). Only 6-aminocaproic acid, muconic acid, and tridecanedioic acid were significantly enriched (Figure 5C). The 2-methylsuccinic acid, glutaric acid and monomethyl succinate were the three compounds with the greatest decline in quantity. Ten long-chain fatty acids, including 2-hydroxyhexadecanoic acid (16:0), 9,10,13-trihydroxy-11-octadecenoic acid (18:1), tetradecanedioic acid (14:0), 12-hydroxyoctadecanoic acid (18:0), crepenynic acid (18:1), methyl palmitate (17:0), myristic acid (14:0), palmitaldehyde (16:0), palmitic acid (16:0), and palmitoleic acid (16:1), were profiled. No overlapping change trends were obtained for these long-chain fatty acids in the two tissues. Only five of the compounds showed the same alteration trend between the two tissues, indicating that the pathogen infection had different metabolic effects on roots and leaves.

**Figure 6:**
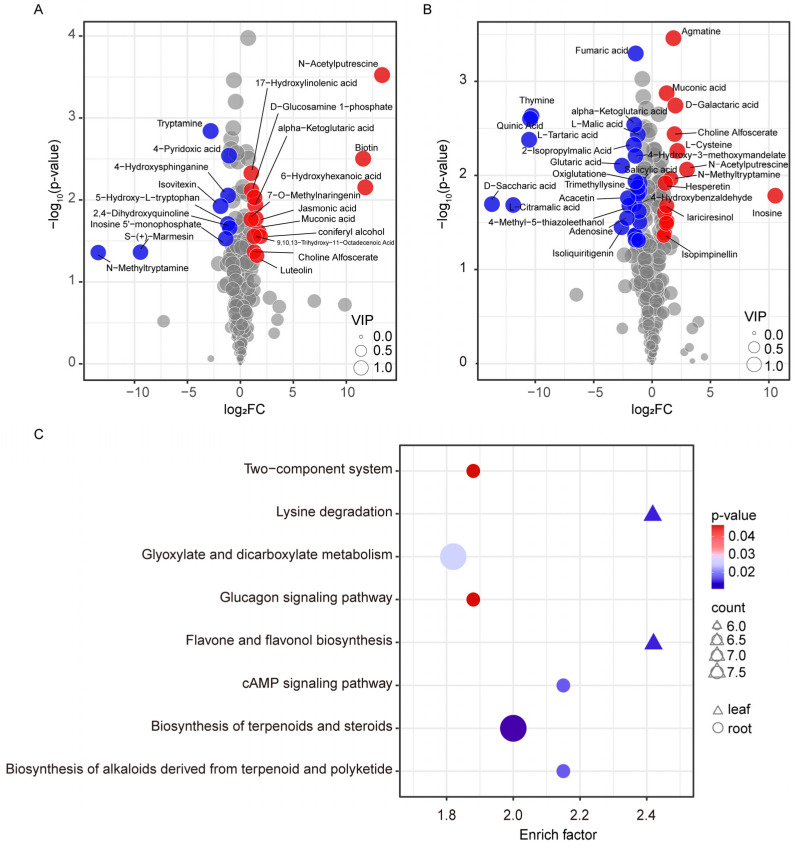
Differentially accumulated metabolites (DAMs) with KEGG pathway information and the enriched pathways of these DAMs. (**A**): DAMs in the leaves after CLas infection; (**B**): DAMs in the roots. The metabolites whose change did not meet the differentially accumulation threshold were filled with gray color in A and B. The upregulated and the downregulated ones were colored in red and blue, respectively. The cycle size indicated the VIP value in the OPLS-DA analysis; (**C**): Pathway enrichment analysis of the DAMs using the KEGG annotation background information detecting in the clusterProfiler software [36]. Only pathways with *p*-value < 0.05 in the Fisher’s exact test were plotted.

We also detected a large number of other metabolites affected by HLB in leaves and roots. After annotating in the KEGG database [34], a total of 243 compounds obtained metabolic pathway information (Table S2, Supplementary Materials). Among these compounds, 67 metabolites were differentially accumulated in the leaf tissues, with 31 being decreased and 36 being increased (Figure 6A). The differential regulation of the content of 97 molecules was discovered in the infected roots; a total of 57 of which declined in contrast to those 40 that were mounted (Figure 6B). These DAMs covered a wide range of the classes of plant metabolites, including organic acids, purine, flavonoids, as well as plant hormone-related substances. The level of jasmonic acid was found to be elevated in the leaves, while the salicylic acid content had declined in the roots. A total of 28 metabolites (out of 136) were found in both of the tissues. Nevertheless, seven of these shared compounds exhibited an opposite alteration. For example, the content of N-methyltryptamine, which was found ubiquitously in the citrus plants for defense against aggressors [35], was declined more than 1000 times in the leaves. In contrast, the content of this chemical in the roots increased to about three times higher than that of the healthy plants. The pathway enrichment analysis indicated that the lysine metabolism, flavonoid and flavonol biosynthesis pathway, were the two that were significantly enriched in the leaves (Figure 6C). In contrast, the biosynthesis of the terpenoids and steroids, the biosynthesis of the alkaloids derived from terpenoid and polyketide, the cAMP signaling pathway, the glyoxylate and dicarboxylate metabolism, the two-component system, and the glucagon signaling pathways were significantly over-represented in the roots (Figure 6C).

## 3. Discussion

HLB could occur in almost all of the commercial citrus cultivar without a cure. However, the responses to the disease varied from variety to variety [13,37,38]. Looking for new resistant, or at least tolerant, plant materials is one direct and efficient approach to cope with the current situation. Here, for the first time we investigated the physiological responses of a new citrus rootstock *C. junos* ‘Pujiang Xiangcheng’ to the invasion by CLas. After 15 weeks of grafting inoculation with CLas, very few of the leaves showed signs of mild disease, but it was obvious that the new flushes were impaired, and the root systems were damaged (Figure 1). A large number of fibrous roots were lost. The recent convincing results using the split root system have demonstrated the significance of root infection for the systematic spread of the pathogen within the trees [10]. The colonization of the bacterium also caused server root impairment prior to the foliar symptoms [6,9]. The root damage was not ascribed to carbohydrate starvation due to phloem-plugging, because the phloem was still working at this stage. As shown in our data (Figure 3), the phloem was only partially plugged due to callus deposition. However, the phloem fibers were dramatically thickened and the infected leaf tissues suffered severe cell necrosis, characterized by misshapen or collapsed phloem cells, manifesting typical anatomic symptoms of HLB reaction as previously observed [12,13]. It has been proposed that the continued growth of the tree due to the regeneration of the impaired phloem was the characteristic of the tolerant cultivars. Although we still do not know whether it holds true for *C. junos* from the current data, it seems unlikely that it harbors an advance in combating the CLas pathogen, because the photosynthetic system was already damaged, as shown in the decline of the photosynthetic pigments (Figure 1), the decreased photosynthetic rate (Figure 2), as well as the accumulation of starch granules in the phloem cells (Figure 3J).

Ma et al. [33] documented the production of reactive oxygen species (ROS) shortly after the initiation of new flushes in the phloem. This reaction positively triggered callose deposition which, in turn, incited symptom development by instigating cell death [39]. The genes coding for the antioxidant enzymes, as well as the redox homeostasis-related proteins, were downregulated in several previously investigated citrus plants [19,33,40]. However, the results for the alternation of activity of the enzymes were inconsistent among the studies. The markedly elevated activities of all three antioxidant enzymes were observed by Pitino et al. [39]. Franco et al. [41] found that in sweet orange Harmlin and N-33, Huanglongbing induced the accumulation of peroxidase transcripts, but POD enzyme activities exhibited different alterations: the enzyme activity was downregulated after infection in Harmlin, while the enzyme activity was upregulated after infection in N-33 [41]. In the tolerant cultivar Kaffir lime, Cu/Zn-SOD and POD4 were upregulated, while they were downregulated in *C. sinensis* [19]. In the current investigation of *Citrus junos*, significant decreases in the CAT and SOD activity were observed. The POD activity did not show any significant change. The superoxide dismutase and catalase are two of the most potent antioxidants in plants for protecting the cells against oxidative stress posed by superoxides or H_2_O_2_. We therefore suggested that these enzymes might be held hostage by the CLas pathogens, and cannot function properly to mitigate the ROS injuries.

There is growing evidence that the host’s primary and secondary metabolism are altered due to the infection by CLas, which are also indicators of the susceptibility or tolerance of the plant resources. Sugars not only provide energy for plants’ growth and defense responses, but also act as signals in modulating plants’ adaptation to internal and external stimuli, including invading pathogens. Several of the previous investigations have revealed that the level of different types of sugars were altered in the HLB-infected tissues, such as leaves, barks, roots, and fruits, but the trend varied depending on the variety, tissues, and disease progression [17,22,30,42,43,44]. Generally, the sugar contents were decreased after infection. Peng et al. [22] explored the metabolomic changes of the Chongyi wild mandarin in the positive- or mock-inoculated plants. The sucrose levels were increased in both the infected leaves and barks, but did not change in the roots. Similar results were observed in the experiment of Freitas et al. [45]. In the results of Albrecht et al. [43], only the fructose and xylose were specifically downregulated in the roots, while raffinose was accumulated in the HLB leaves. Interestingly, the sugar alterations were coordinated with the disease’s progress, based on the longitudinal experimental designs [17]. Albrecht et al. [43] observed that the glucose and fructose content were increased eight months after inoculation with CLas, but these compounds were significantly decreased at the time of 10 mai. These dynamic changes could reflect the pathogenesis of CLas during the invasion. In our case, 15 wai, most of the detected sugars were downregulated in the roots and leaves, including the lactobiose, isomaltulose, D-galactose, and the D-(−)-threose, indicating that these carbohydrates were indirectly consumed by either the pathogen for replication or by the plant itself in the defending reaction, which was in line with the results of Albrecht et al. [43]. Isomaltulose is a structural isomer of sucrose, which can be actively catabolized by the plant’s sugar catabolic enzymes [46]. Although it has been commonly used as a sweetening agent in the food industry, its biofunction in plants still lacks any deep investigation. The intermediates of the biosynthesis of starch, including the glucose 1-phosphate and the glucose 6-phosphate in the roots, were significantly decreased, but this phenomenon was not observed in the leaf tissues (Figure 5A). In contrast, the maltotrioses were dramatically augmented. Considering that the phloem system was still functional at this timepoint (Figure 3), the observed decline in the substrate for starch synthesis could be explained by the metabolite flow into starch accumulation in the roots, as with those found by Xie et al., and Fan et al. [12,47]. One of the very interesting phenomena found here was the accumulation of maltotriose, which was recognized as the indicator of the starch debranching process [48]. This implies that besides the starch synthesis, an active break down of the starch in the roots was also occurring. The intermediate compound of the glycolytic pathway fructose-1,6-biphosphate was uniquely accumulated in the leaves of HLB (Figure 5A). In combination with the results that almost all of the detected products of the downstream tricarboxylic acid were repressed (Figure 5B), we speculated that at this time-point (asymptomatic stage), the regular energy producing networks were already rewired by the pathogen.

A total of five and seven amino acids were detected to be differentially accumulated in the leaves and roots of *C. junos*, respectively, due to HLB. The leucines and isoleucines were altered with the upregulating trend in both of the tissues, while glutamic acid and methionine were decreased regardless of tissue types. These reactions to the CLas were exactly same as those found in the Valencia sweet orange (*C. sinensis*) [49]. The common increase in the prolines in the susceptible citrus plants [43] was not observed (*p* = 0.69, FC = 1.05, VIP = 0.29) in this study. Proline was recognized as one amino acid playing an indispensable role for plants in maintaining the regular growth under abiotic or biotic stress [50]. It remains unknown when this active defense reactivity initiated in the battle between the pathogens and the citrus plants.

Fatty acids, especially the long-chain fatty acids (FA), were obviously decreased in the three different citrus cultivars after being naturally infected with CLas [51,52]. In our results, forty-two fatty acids and conjugates were detected. It is very interesting to find that opposite change trends were manifested in the leaves and roots (Figure 5C). More than a half (61.5%) of these FA were upregulated in the infected leaves, while over 80% were downregulated in the roots. The fatty acid changes have been shown to be able to discriminate the trees of infected or healthy status, according to the results of Suh et al. [52]. They are important building blocks of the plant cell. They also act as the signal modulators in the plant’s defense against diversities of stress by their involvement in the production of reactive oxygen species [53]. The depletion of most FA in the roots were positively related to the observed necrotic and dysfunction fibrous roots, as the integrity of the cell wall were severely impaired (Figure 1). A further study of the change dynamics of the detected FA in the leaves would provide useful information for understanding the interaction between the host and the pathogen.

The metabolic pathways of 243 metabolites were obtained through KEGG pathway annotation (Table S2, Supplementary Materials) in this report. These compounds were found in the leaves for lysine metabolism, flavonoid and flavonol biosynthesis, tropane, piperidine and pyrimidine biosynthesis, shikimate pathway alkaloid synthesis, and porphyrin and chlorophyll metabolic pathways. Meanwhile, those identified in the roots mainly fall into the categories of terpenes, terpenoid and polyketide-derived alkaloid synthesis, cAMP signaling pathway, glyoxylic acid, and dicarboxylate metabolic pathways. Almost consistent results were observed that the secondary metabolic pathways, such as the flavonoid and flavonol biosynthesis, terpenoid and alkaloid biosynthesis, were activated during the progress of HLB, regardless of the stages in the previous investigations of HLB-related physiological and biochemical studies [28,29,54,55,56]. Our results confirmed that *C. junos* was also armed with these tools to defend against the invasion of the CLas pathogen. Nonetheless, the responses of the plants’ system-resistance mediator salicylic and jasmonic acid hormone varied from variety to variety. An important speculation was that the HLB tolerant resources sustained ongoing growth at the expense of reducing defense [57]. In the current explored *C. junos*, the level of jasmonic acid was found to be elevated in the leaves, while the salicylic acid content declined in the roots. Consistent with these results, salicin, a precursor to salicylic acid, accumulated in the leaves of CLas positive (FC = 0.329, *p* = 0.02), which was similar to the results of Albrecht et al. [43]. These results indicated that *C. junos* did not compromise the usage of the systematic defense tool, which brought further metabolite alterations, as shown in Figure 6. This phenomenon was not observed in the root system, which further reflected the different reactions between the roots and leaves.

## 4. Materials and Methods

### 4.1. Plant Materials

The seeds of the wild ‘Pujiang Xiangcheng’ (*C. junos*) were collected in Pujiang County, Sichuan province, China, in October 2018. The one-year-old seedlings were planted in 50 cm diameter containers in December 2019. The growing substrate consisted of field soil, peat moss, and perlite in a volume ratio 6:3:1. A slow-release fertilizer (15:15:15) was added. The plants were watered once a week. In April 2020, all of the plants were moved into a greenhouse with a 200-mesh insect-proof net for the inoculation experiments. The plants were heavily pruned to promote new flushes. Thirty plants were grafted with budwoods which were CLas positive (kindly provided by Dr. Xuefeng Wang, Citrus Research Institute of Chinese Academy of Agricultural Sciences, Chongqing, China). The position of the grafting on the main trunk was chosen ~10 cm above the ground. Another 30 plants of the same size and similar growth status served as the controls by grafting with healthy budwood of CLas negative plants. All of the plants were cultured in the same greenhouse, but separated in an insect-proof net room to keep the growing conditions the same. The greenhouse had no environmental control apparitors, so the plants were grown under natural light and temperature conditions. The symptoms of the leaves were observed at a fixed time every month. Three months after inoculation, the new leaves of the flush were collected for the extraction of genomic DNA. Three leaves of each plant were collected and stored at −20 °C until use. Ten CLas positive plants with a similar tiller as detected by real-time quantitative PCR (RT-qPCR) were chosen for physiological and anatomic analysis. For the metabolomic sampling, three plants with the same growth vigor were chosen. Three mature and three young leaves from each of the three plants were pooled and snap-frozen in liquid nitrogen. The fibrous roots were collected from the same three plants. They were washed in di-distilled water to get rid of the soil, and rinsed once before storing at −80 °C. Three biological replicates were conducted.

### 4.2. Quantification of CLas Pathogen Using RT-qPCR

The leaf blade and the main vein were separated from the leaves for the DNA isolation, using a new razor blade for each sample. The genomic DNA of these leaf materials was extracted by using a modified CTAB method [58]. Briefly, 100 mg of leaf tissue ground in liquid nitrogen was added into a 1.5 mL Eppendorf tube. The cells were lysed in a DNA extraction buffer (3% CTAB, 200 mM Tris-HCl, 50 mM Na_2_EDTA, 1.4 M NaCl) at 60 °C for 45 min. The lysate was extracted twice using chloroform. The nucleic acids were precipitated in the presence of 0.3 M sodium acetate and 50% of isopropanol. The integrity and purity of the DNA were determined by conventional agarose gel electrophoresis, and UV spectrum (280 and 260 nm) by using Nanodrop 2000 (Thermo, Wilmington, DE, USA).

RT-qPCR was employed to detect the infection status of CLas in each seedling. A recombinant plasmid containing a fragment of 16S rDNA of CLas was constructed (File S1, Supplementary Materials) to serve as the quantification reference. A standard two-step qPCR protocol was adopted. Forty-five cycles of PCR reaction were carried out (95 °C for 5 s; 60 °C for 30 s). The PCR mixture was set up as follows: 25 ng DNA (sample or reference plasmid); 10 pmol of qHLBas and qHLBr primer (Table S4, Supplementary Materials); 10 μL of TB Green Premix (TaKaRa, Dalian, China); H_2_O to a total volume of 20 μL. A standard curve was generated using the same amplification system, using the serial dilutions of recombinant plasmid DNA described above. Each run contained three technical replicates in the same PCR plate. The plants were considered to be CLas-positive when the cycle threshold value was below 37, as indicated by the standard curve.

### 4.3. Leaf Photosynthesis Measurements

The photosynthetic parameters of the CLas-infected plants as well as the control plants were measured using a portable photosynthesis meter (LI-6800XT, LI-COR Biosciences, Lincoln, NE, USA) three months and a half after inoculation. The assay was performed between 8:30 to 10:30 AM in the sunny mornings. The parameters, such as net photosynthesis rate (Pn), transpiration rate (Tr), stomatal conductance (Gs), and intercellular CO_2_ concentration (Ci), were measured and recorded. For each measurement, three plants were selected. Three expanded mature leaves in the upper third lateral branches were chosen. The water utilization efficiency (WUE, Pn/Tr × 100%) and the instantaneous carboxylation efficiency (MCE, Pn/Ci × 100%) of mesophyll were also calculated. The average values of the duration were used for the photosynthesis efficiency estimation.

### 4.4. The Anatomy Microstructure of the Vascular Bundle of Leaf

The leaf midrib tissues of both the healthy and HLB plants were used for observations. The tissue fixation, embedding, and sectioning were completed with reference to the protocol of Deng et al. [13]. The midrib segments, 0.5 cm in length, were fixed in the FAA fixative solution at room temperature for at least 24 h. The dehydration of the specimens was achieved by immersion in 70%, 85%, and 95% ethanol sequentially each for 2 h. Finally, 100% ethanol was used twice, one for 2 h and the other for 12 h at room temperature. The ethanol was gradually replaced with xylene using one hour treatment in solutions that consisted of 1:2, 1:1, 2:1 to 0:1 (*v*/*v*) of ethanol: xylene. The samples were kept in xylene solution for another 12 h before paraffin infiltration. To enhance the infiltration effect of paraffin, the tissues were first placed in the mixture of melted paraffin wax and xylene (1:1, *v*/*v*) in a tightly closed container for incubating at 45 °C overnight. The temperature was raised to 50 °C and kept for 3 h, then to 55 °C for 2 h, and finally to 60 °C for 1 h. The solution was replaced with pure paraffin wax. The solution change was repeated twice every 2 h before maintaining at 60 °C overnight. The tissues were embedded in 100% paraffin using the embedding mold with the desired orientation at room temperature. The molds were kept on ice to facilitate the hardening process. The blocks were then sectioned on a microtome (YL3 rotary; Shanghai, China) with the section thickness set to 8 μm. The sections were floated on a drop of hot water (40 °C) on a microscope slide and placed on a hot plate at 40 °C overnight to let the section adhere firmly to the slides.

The slides were dewaxed with pure xylene for 2 h and passed through incubating in the xylene/ethanol solution at the ratio of 3:1, 1:3 (*v*/*v*), respectively, for 2 min. The rehydration of the cells was achieved by passing through a series of decreasing ethanol washes (100% 2 min, 95% 2 min, 85% 2 min, 70% 2 min, 50% 2 min, and 35% 2 min). The tissues were initially stained with 1% safranin-O for 2 h. Afterwards, an increasing ethanol wash was carried out (35%, 50%, 70%, 85%, 95%, 2 min per wash). The slides were counterstained with 0.5% fast green for 30 s. The slices were rinsed, using 95% ethanol for 2 min, and finally 100% ethanol for 2 × 1 min. When the starch grains were to be stained, methylene blue-azure II-basic fuchsin [59] was used. When the callose deposition in the leaves was investigated, the slides were stained with aniline blue for one minute and directly observed under an epifluorescence microscope. Otherwise, the xylene was used to clear the cells before mounting with neutral balsam. The slides were observed under an Olympus BX51 microscope with a U-MWU2 filter cube.

### 4.5. Determination of Antioxidant Enzyme Activities in ‘Pujiang Xiangcheng’ Infected with CLas

The activities of SOD, POD, and CAT were determined using the following photochemical methods. The CAT enzyme activity was measured referring to the protocol of Kato and Shimiz [60]. Briefly, 100 mg of the fine-powdered tissue was extracted using 0.2 mL K_2_HPO_4_/KH_2_PO_4_ buffer pH7.8 at 4 °C for 30 min. The supernatant (100 μL) was collected via centrifugation at 4 °C for 10 min. On the ice, 2.6 mL of the buffer, 0.3 mL 0.1 M H2O2, was added to the supernatant. The absorbance was recorded at 240 nm for 40 min on a spectrophotometer (Multiskan GO, Thermal, Wilmington, DE, USA), with the chamber temperature being kept at 30 °C. One enzyme unit was defined as a reduction of absorbance at 240 nm of 0.01 per minute. The SOD activity was determined after the methods of Cheng et al. [61]. Two hundred microliter of 0.05 M K_2_HPO_4_/KH_2_PO_4_ buffer (pH 7.8) were used to extract the enzyme proteins in 100 mg plant material at 4 °C for 30 min. After centrifuging, 100 μL supernatant was kept. The riboflavin, nitroblue tetrazolium (NBT), methionine, and ethylenediamine tetra-acetic sodium were sequentially added to the extract to a final concentration of 2 µM, 65 µM, 13 µM, and 1 µM, respectively, in the same buffer. The solution was transferred to a glass tube. The mixture was illuminated (4000 lx) at 25 °C for 20 min. The absorbance of blue formazan was measured at 560 nm. The amount of protein that caused the 50% NBT reduction was defined as one unit of SOD activity. The POD activity was assessed, following the procedure of Ranieri et al. [62]. The enzymes were extracted from 100 mg leaf powders in 200 μL of sodium phosphate buffer (50 mM, pH 5.7) at 4 °C for 30 min. The lysis was clarified by centrifugation at 4 °C for 10 min. A 30-µL volume of the enzyme solution was added to 2.97 mL of sodium phosphate buffer (50 mM, pH 5.7), containing 5 mM guaiacol and 3.7 mM H_2_O_2_ as the substrates. The increase in absorbance at 470 nm due to the guaiacol oxidation was monitored in 60 s-intervals for 3 min. One unit of peroxidase activity was defined as the amount of enzyme that increased 0.01 of the 470 nm absorbance per minute.

### 4.6. Extraction and Detection of the Metabolites in the Leaves and Roots

To systematically investigate the physiological response of ‘Pujiang Xiangcheng’ to the CLas infection, ultra-performance liquid chromatography–tandem mass spectrometry (UPLC-MS/MS) was used to analyze a widely targeted metabolome in the leaves and roots of CLas+ and the corresponding negative controls. The collected samples were freeze-dried in a vacuum freeze dryer, and then crushed using a mixer mill (MM 400, Retsch GmbH, Haan, Germany) equipped with zirconia beads for 2 min at 30 Hz. One hundred microgram of the resulting powder was extracted in 1.2 mL of 70% methanol through vertexing for 30 min at room temperature, and then at 4 °C overnight with agitation. After centrifugation at 12,000 rpm, the supernatant was transferred and filtered through a 0.22 μm filter prior for UPLC-MS/MS analysis.

The metabolites were separated and characterized on a platform comprised of an UPLC (SHIMADZU Nexera X2 (Kyoto, Japan), equipped with an Agilent SB-C18 column (1.8 µm, 2.1 mm × 100 mm)) and a triple quadrupole-linear ion trap (LIT) mass spectrometer (Applied Biosystems 4500 Q TRAP UPLC-MS/MS). The mobile phase used consisted of phase A: ultrapure water with 0.1% formic acid, and phase B: acetonitrile with 0.1% formic acid. A gradient elution program was used: 5% of B at 0 min; phase B increased linearly to 95% within 9.0 min; 95% B maintained for 1.0 min; 10.0 to 11.1 min, the proportion of phase B was reduced to 5%; 11.1 min to 14.0 min, 5% phase B. The flow rate was set to 0.35 mL/min. The column oven was kept at 40 °C. The injection volume was 4 μL.

The LIT and triple quadrupole (QQQ) scans were acquired with an ESI turbo ion-spray interface controlled by the Analyst software (v1.6.3, AB Sciex, Darmstadt, Germany) in both the positive and negative ion modes. The ESI source operating parameters included: turbo spray ion source; temperature 550 °C; the ion spray voltage 5500 V for positive ion mode and −4500 V for negative ion mode; the ion source gas I, gas II and curtain gas were set to 50, 60 and 25.0 psi, respectively; The collision-induced ionization parameter was set to high. The polypropylene glycol solutions at 10 and 100 μM were used to tune and calibrate the instrument in QQQ and LIT modes, respectively. The multiple reaction monitoring (MRM) was used in the QQQ scan with the collision gas being set to medium. The declustering potential (DP) and collision energy (CE) were optimized for individual MRM transitions. A specific set of MRM transitions was monitored, based on the metabolites eluted during the period.

### 4.7. Qualitative and Quantitative Determination of the Metabolites

The qualitative and quantitative analysis of the metabolites was conducted, following the methods of Yang et al. [63]. Based on a self-established database (Biomarker Technologies Corporation Co., Ltd., Beijing, China) and public databases (MassBank [64], KNApSAcK [65], HMDB [66], MoTo DB [67], and METLIN [68]), the obtained spectrometry data were subjected to qualitative analysis. Prior to the matching of the precise secondary mass spectrometry data, duplicate signals of K^+^, Na^+^, and NH_4_^+^ ions and the isotope signals were excluded. The quadrupole filter selected the required characteristic fragment ions and eliminated the interference of non-target ions. The metabolites were identified by comparing the accurate precursor ion (Q1) and production (Q3) values, the retention time, and the fragmentation pattern with the collected databases, which includes the self-established database and public databases. For the self-established database, it was constructed based on the standard materials and purified compounds. Due to the commercial conflict of interest, the internal database was not publicly available but could be consulted via direct contact (Biomarker Technologies Corporation Co., Ltd., Beijing, China). The MS/MS data were annotated using the tool: Metlin [68] and HMDB [66] with searching parameters: Parent Ion Tolerance 15; MS/MS tolerance 0.1 for Metlin and 0.8 for HMDB; Acceptance Criteria >40 for Metlin and >0.4 for HMDB. The obtained substances were further annotated with the KEGG database [34] and LipidMAP database [69], respectively, using the PubChem identifiers. After obtaining the metabolite spectrum analysis data of the different samples, the peak area integration was performed of all of the substances using the MultiQuant software (v3.0.3, SCIEX, Framingham, MA, USA). The integration correction was performed on the mass spectral peaks of the same metabolite in the different samples. The exported raw data are available at https://doi.org/10.6084/m9.figshare.20485947.v1 (for the negative detection mode) and https://doi.org/10.6084/m9.figshare.20485941.v1 (for the positive detection mode).

### 4.8. Data Analysis

Before conducting the differential analysis of the metabolomic data, unsupervised PCA was used for dimension reduction. The signal intensities of the metabolites were normalized, using unit variance scaling. The first two PCs were selected to judge the degree of variation between the samples with the ‘prcomp’ function in the R package. Furthermore, hierarchical clustering (cor = “pearson”) was conducted after a generalized log transformation of the integral metrology data of all of the metabolites, using the LMgene package [70]. The data were log transformed (log2) and mean-centered before conducting the OPLS-DA. The ropls package (v1.26.4) [71] was used to perform the OPLS-DA, specifying the quantitative data of the metabolites as the X matrix, and the sample grouping (leaf-healthy, leaf-infected, root-healthy, root-infected) as the Y matrix. Cross-validation of the reliability of the OPLS-DA model was judged through the R^2^Y and Q^2^ value, with R^2^Y > 0.5 and Q^2^ > 0.5 being accepted. Based on this model, the VIP values were extracted. Finally, the FC between the different groups, the *p* value of a *t*-test, and the VIP value of the OPLS-DA were used to screen the DAMs between the groups. In this experiment, the screening criteria were defined as |FC| > 1, *p* < 0.05, and VIP > 1.

The pathway enrichment analysis was performed using the clusterProfiler (v4.2.2) R package [36] against the background of the annotated pathway information in the KEGG [34] and LipidMAP [69] database. Significantly enriched metabolic pathways were judged with a *p*-value < 0.05. The analysis of variance of the other data was performed using the SPSS software (v22, IBM, Armonk, NY, USA) or the R stat functions. One-way ANOVA was used. The significance thresholds were chosen as *p* < 0.05 (*), *p* < 0.01 (**). Tukey’s range test was used when multiple comparisons were involved. Linear regression fitting was performed using EXCEL 2020 software (Microsoft, Redmond, WA, USA). The plots were drawn using SigmaPlot (Systat Software, Inc., San Jose, CA, USA), and the R package ggplot2.

## 5. Conclusions

For the first time, we analyzed the physiological and metabolomic responses of *C. junos* to the HLB infection in the leaf and root tissues, revealing the susceptibility of the plants under the tested condition. The sugars, amino acids, fatty acids, as well as several secondary metabolism products were significantly disturbed by the invasion of the pathogen. Specifically, the depletion of the D-(−)-threose, isomaltulose, glutamic acid, and methionine; the increase in the leucine and isoleucine could be viewed as the biomarkers for the indication of HLB infection in *C. junos*. Our results also revealed the obvious distinct reaction to the pathogen in the roots and leaves, especially for the fatty acids’ metabolism, and several other KEGG pathways, which highlighted the importance of root resistance in the interaction of host–pathogen of CLas.

## Data Availability

Not applicable.

## Supplementary Materials

The following supporting information can be downloaded at: https://www.mdpi.com/article/10.3390/ijms23169242/s1.
