# Supplementary material for: Metabolomic Analysis Revealed Distinct Physiological Responses of Leaves and Roots to Huanglongbing in a Citrus Rootstock
Source: Int J Mol Sci. 2022 Aug 17;23(16):9242. doi: 10.3390/ijms23169242 (PMC9409271; doi:10.3390/ijms23169242)
Supplement: Supplementary file 1 [file ijms-23-09242-s001.zip › Supplementary Figure S1.pdf]

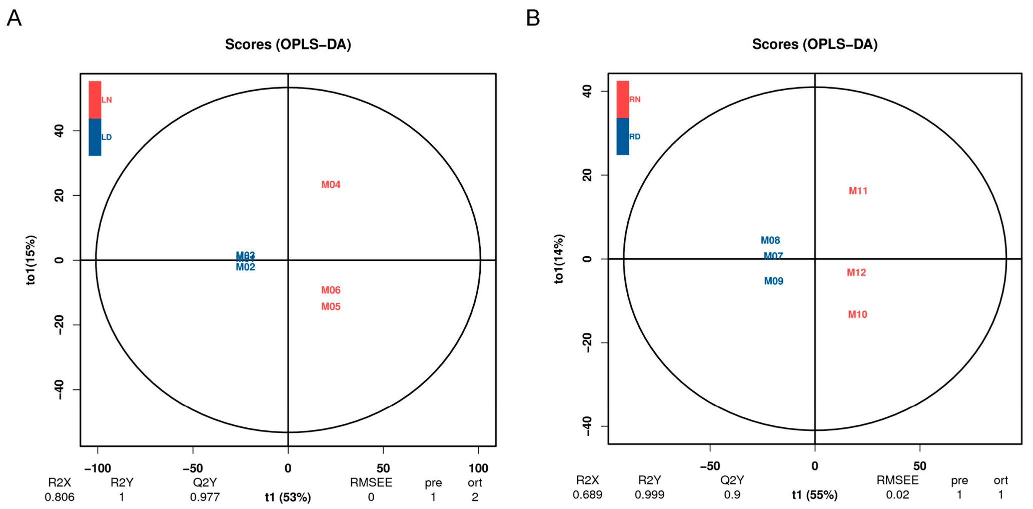

Supplementary Figure S1 OPLS-DA model score plot. A: discriminant model scores for the infected leaves; B: discriminant model score between the healthy group and the Huanglongbing infection group in roots. $Q^2 > 0.5$ can be considered as an effective model, and $Q^2 > 0.9$ is an excellent model. M01-M03: diseased leave sample 1 to sample 3; M04-M06: normal leave samples; M07-M09: diseased root samples; M10-M12: healthy root tissues.
