# Supplementary material for: Metabolomic Analysis Revealed Distinct Physiological Responses of Leaves and Roots to Huanglongbing in a Citrus Rootstock
Source: Int J Mol Sci. 2022 Aug 17;23(16):9242. doi: 10.3390/ijms23169242 (PMC9409271; doi:10.3390/ijms23169242)
Supplement: Supplementary file 1 [file ijms-23-09242-s001.zip › Supplementary File S1.pdf]

To construct the standard plasmid for absolute quantification of CLas in the samples, a fragment of 16S rRNA were amplified using primers HLBas-R and HLBas-F. The PCR mixture used was: 50 ng of total DNA from CLas-positive budwood, 10 pmol of upstream and downstream primer, 10 μL of high-fidelity DNA polymerase/dNTP master mix, H_2O to a total volume of 20 μL. The amplicons were ligated to the pEASY-blunt vector (Transgen, Beijing, China) referring to the cloning kit. The recombinant plasmid was transformed into Escherichia coli Trans-T1 by heat shock method. The transformed strains were screened by the pressure of 50 mg/L kanamycin. Single colonies were picked for culture. Plasmid were extracted using a plasmid mini-kit from Shanghai Sangon Bioengineering Co., Ltd. Plasmids were sequenced.

Plasmid information:

```
LOCUS       Exported                4267 bp ds-DNA     circular SYN 10-MAR-2022
DEFINITION  synthetic circular DNA
ACCESSION   .
VERSION     .
KEYWORDS    .
SOURCE      synthetic DNA construct
  ORGANISM  synthetic DNA construct
REFERENCE   1  (bases 1 to 4267)
  AUTHORS   CCQQ
  TITLE     Direct Submission
  JOURNAL   Exported Jun 13, 2022 from SnapGene 4.2.4
            http://www.snapgene.com
FEATURES             Location/Qualifiers
     source          1..4267
                     /organism="synthetic DNA construct"
                     /mol_type="other DNA"
     CDS             1..213
                     /codon_start=1
                     /gene="lacZ fragment"
                     /product="LacZ-alpha fragment of beta-galactosidase"
                     /label=lacZ-alpha

/translation="KGQFAAAKFNSPYSESYYNSLAVVLQRRDWENPGVTQLNRLAAHP
                     PFASWRNSEEARTDRPSQQLRSLYVR"
     promoter        complement(35..53)
                     /label=T7 promoter
                     /note="promoter for bacteriophage T7 RNA polymerase"
     primer_bind     complement(60..76)
                     /label=M13 fwd
                     /note="common sequencing primer, one of multiple similar
                     variants"
```

CDS 214..516
/codon_start=1
/gene="ccdB"
/product="CcdB, a bacterial toxin that poisons DNA gyrase"
/label=ccdB
/note="Plasmids containing the ccdB gene cannot be propagated in standard E. coli strains."
/translation="QFKVYTYKRESRYRLFVDVQSDIIDTPGRRMVIPLASARLLSDKV

SRELYPVVHIGDESWRMMTTDMASVPVSVIGEEVADLSHRENDIKNAINLMFWGI"

CDS 865..1659
/codon_start=1
/gene="aph(3')-II (or nptII)"
/product="aminoglycoside phosphotransferase from Tn5"
/label=NeoR/KanR
/note="confers resistance to neomycin, kanamycin, and G418 (Geneticin(R))"

/translation="MIEQDGLHAGSPAAWVERLFGYDWAQQTIGCSDAAVFRLSAQGRP

VLFVKTDLSGALNELQDEAARLSWLATTGVPCAAVLDVVTEAGRDWLLLGEVPGQDLLS

SHLAPAEKVSIMADAMRRLHTLDPATCPFDHQAKHRIERARTRMEAGLVDQDDLDEEHQ

GLAPAELFARLKASMPDGEDLVVTHGDACLPNIMVENGRFSGFIDCGRLGVADRYQDIA

LATRDIAEELGGEWADRFLVLYGIAAPDSQRIAFYRLLDEFF"

CDS complement(1909..2769)
/codon_start=1
/gene="bla"
/product="beta-lactamase"
/label=AmpR
/note="confers resistance to ampicillin, carbenicillin, and related antibiotics"

/translation="MSIQHFRVALIPFFAAFCLPVFAHPETLVKVKDAEDQLGARVGYI

ELDLNSGKILESFRPEERFPMMSTFKVLLCGAVLSRIDAGQEQLGRRIHYSQNDLVEYS

PVTEKHLTDGMTVRELCSAAITMSDNTAANLLLTTIGGPKELTAFLHNMGDHVTRLDRW

EPELNEAIPNDERDTTMPVAMATTLRKLLTGELLTLASRQQLIDWMEADKVAGPLLRSA

LPAGWFIADKSGAGERGSRGIIAALGPDGKPSRIVVIYTTGSQATMDERNRQIAEIGAS

LIKHW"

```
     rep_origin      2893..3481
                     /direction=RIGHT
                     /label=ori
                     /note="high-copy-number ColE1/pMB1/pBR322/pUC origin of
                     replication"
     promoter        3805..3835
                     /label=lac promoter
                     /note="promoter for the E. coli lac operon"
     protein_bind    3843..3859
                     /label=lac operator
                     /bound_moiety="lac repressor encoded by lacI"
                     /note="The lac repressor binds to the lac operator to
                     inhibit transcription in E. coli. This inhibition can be
                     relieved by adding lactose or
                     isopropyl-beta-D-thiogalactopyranoside (IPTG)."
     primer_bind     3867..3883
                     /label=M13 rev
                     /note="common sequencing primer, one of multiple similar
                     variants"
     CDS             3879..3956
                     /codon_start=1
                     /gene="lacZ fragment"
                     /product="LacZ-alpha fragment of beta-galactosidase"
                     /label=lacZ-alpha
                     /translation="MTMITPSSELTLTKGTSPAGLNELAL"
     promoter        3904..3922
                     /label=T3 promoter
                     /note="promoter for bacteriophage T3 RNA polymerase"
     primer_bind     3957..3977
                     /label=HLBas-F
     primer_bind     3975..3994
                     /label=HLBas
     primer_bind     complement(4026..4049)
                     /label=HLBr
     primer_bind     complement(4244..4267)
                     /label=HLBas-R
ORIGIN
        1 aagggccaat tcgcggccgc taaattcaat tcgccctata gtgagtcgta ttacaattca
       61 ctggccgtcg ttttacaacg tcgtgactgg gaaaaccctg gcgttaccca acttaatcgc
      121 cttgcagcac atccccctttt cgccagctgg cgtaatagcg aagaggcccg caccgatcgc
      181 ccttcccaac agttgcgcag cctatacgta cggcagttta aggtttacac ctataaaaga
      241 gagagccgtt atcgtctgtt tgtggatgta cagagtgata ttattgacac gccggggcga
      301 cggatggtga tccccctggc cagtgcacgt ctgctgtcag ataaagtctc ccgtgaactt
      361 tacccggtgg tgcatatcgg ggatgaaagc tggcgcatga tgaccaccga tatggccagt
```

421 gtgccggtct ccgttatcgg ggaagaagtg gctgatctca gccaccgcga aaatgacatc
481 aaaaacgcca ttaacctgat gttctgggga atataaatgt caggcatgag attatcaaaa
541 aggatcttca cctagatcct tttcacgtag aaagccagtc cgcagaaacg gtgctgaccc
601 cggatgaatg tcagctactg ggctatctgg acaagggaaa acgcaagcgc aaagagaaag
661 caggtagctt gcagtgggct tacatggcga tagctagact gggcggtttt atggacagca
721 agcgaaccgg aattgccagc tggggcgccc tctggtaagg ttgggaagcc ctgcaaagta
781 aactggatgg ctttcttgcc gccaaggatc tgatggcgca ggggatcaag ctctgatcaa
841 gagacaggat gaggatcgtt tcgcatgatt gaacaagatg gattgcacgc aggttctccg
901 gccgcttggg tggagaggct attcggctat gactgggcac aacagacaat cggctgctct
961 gatgccgccg tgttccggct gtcagcgcag gggcgcccgg ttctttttgt caagaccgac
1021 ctgtccggtg ccctgaatga actgcaagac gaggcagcgc ggctatcgtg gctggccacg
1081 acgggcgttc cttgcgcagc tgtgctcgac gttgtcactg aagcgggaag ggactggctg
1141 ctattgggcg aagtgccggg gcaggatctc ctgtcatctc accttgctcc tgccgagaaa
1201 gtatccatca tggctgatgc aatgcggcgg ctgcatacgc ttgatccggc tacctgccca
1261 ttcgaccacc aagcgaaaca tcgcatcgag cgagcacgta ctcggatgga agccggtctt
1321 gtcgatcagg atgatctgga cgaagagcat caggggctcg cgccagccga actgttcgcc
1381 aggctcaagg cgagcatgcc cgacggcgag gatctcgtcg tgacccatgg cgatgcctgc
1441 ttgccgaata tcatggtgga aaatggccgc ttttctggat tcatcgactg tggccggctg
1501 ggtgtggcgg accgctatca ggacatagcg ttggctaccc gtgatattgc tgaagagctt
1561 ggcggcgaat gggctgaccg cttcctcgtg ctttacggta tcgccgctcc cgattcgcag
1621 cgcatcgcct tctatcgcct tcttgacgag ttcttctgaa ttattaacgc ttacaatttc
1681 ctgatgcggt attttctcct tacgcatctg tgcggtattt cacaccgcat caggtggcac
1741 ttttcgggga aatgtgcgcg gaacccctat ttgtttattt ttctaaatac attcaaatat
1801 gtatccgctc atgagattat caaaaaggat cttcacctag atccttttaa attaaaaatg
1861 aagttttaaa tcaatctaaa gtatatatga gtaaacttgg tctgacagtt accaatgctt
1921 aatcagtgag gcacctatct cagcgatctg tctatttcgt tcatccatag ttgcctgact
1981 ccccgtcgtg tagataacta cgatacggga gggcttacca tctggcccca gtgctgcaat
2041 gataccgcga gacccacgct caccggctcc agatttatca gcaataaacc agccagccgg
2101 aagggccgag cgcagaagtg gtcctgcaac tttatccgcc tccatccagt ctattaattg
2161 ttgccgggaa gctagagtaa gtagttcgcc agttaatagt ttgcgcaacg ttgttgccat
2221 tgctacaggc atcgtggtgt cacgctcgtc gtttggtatg gcttcattca gctccggttc
2281 ccaacgatca aggcgagtta catgatcccc catgttgtgc aaaaaagcgg ttagctcctt
2341 cggtcctccg atcgttgtca gaagtaagtt ggccgcagtg ttatcactca tggttatggc
2401 agcactgcat aattctctta ctgtcatgcc atccgtaaga tgcttttctg tgactggtga
2461 gtactcaacc aagtcattct gagaatagtg tatgcggcga ccgagttgct cttgcccggc
2521 gtcaatacgg gataataccg cgccacatag cagaacttta aaagtgctca tcattggaaa
2581 acgttcttcg gggcgaaaac tctcaaggat cttaccgctg ttgagatcca gttcgatgta
2641 acccactcgt gcacccaact gatcttcagc atcttttact ttcaccagcg tttctgggtg
2701 agcaaaaaca ggaaggcaaa atgccgcaaa aaagggaata agggcgacac ggaaatgttg
2761 aatactcata ctcttccttt ttcaatatta ttgaagcatt tatcagggtt attgtctcat
2821 gaccaaaatc ccttaacgtg agttttcgtt ccactgagcg tcagaccccg tagaaaagat
2881 caaaggatct tcttgagatc ctttttttct gcgcgtaatc tgctgcttgc aaacaaaaaa
2941 accaccgcta ccagcggtgg tttgtttgcc ggatcaagag ctaccaactc tttttccgaa
3001 ggtaactggc ttcagcagag cgcagatacc aaatactgtt cttctagtgt agccgtagtt

3061 aggccaccac ttcaagaact ctgtagcacc gcctacatac ctcgctctgc taatcctgtt

3121 accagtggct gctgccagtg gcgataagtc gtgtcttacc gggttggact caagacgata

3181 gttaccggat aaggcgcagc ggtcgggctg aacggggggt tcgtgcacac agcccagctt

3241 ggagcgaacg acctacaccg aactgagata cctacagcgt gagctatgag aaagcgccac

3301 gcttcccgaa gggagaaagg cggacaggta tccggtaagc ggcagggtcg gaacaggaga

3361 gcgcacgagg gagcttccag ggggaaacgc ctggtatctt tatagtcctg tcgggtttcg

3421 ccacctctga cttgagcgtc gatttttgtg atgctcgtca ggggggcgga gcctatggaa

3481 aaacgccagc aacgcggcct ttttacggtt cctggccttt tgctggcctt ttgctcacat

3541 gttctttcct gcgttatccc ctgattctgt ggataaccgt attaccgcct ttgagtgagc

3601 tgataccgct cgccgcagcc gaacgaccga gcgcagcgag tcagtgagcg aggaagcgga

3661 agagcgccca atacgcaaac cgcctctccc cgcgcgttgg ccgattcatt aatgcagctg

3721 gcacgacagg tttcccgact ggaaagcggg cagtgagcgc aacgcaatta atgtgagtta

3781 gctcactcat taggcacccc aggctttaca ctttatgctt ccggctcgta tgttgtgtgg

3841 aattgtgagc ggataacaat ttcacacagg aaacagctat gaccatgatt acgccaagct

3901 cagaattaac cctcactaaa gggactagtc ctgcaggttt aaacgaattg gcccttaggc

3961 ctaacacatg caagtcgagc gcgtatgcaa tacgagcggc agacgggtga gtaacgcgta

4021 ggaatctacc tttttctacg ggataacgca tggaaacgtg tgctaatacc gtatacgccc

4081 tattggggga aagattttat tggagagaga tgagcctgcg ttggattagc tagttggtag

4141 ggtaagagcc taccaaggct acgatctata gctggtctga gaggacgatc agccacactg

4201 ggactgagac acggcccaga ctcctacggg aggcagcagt ggggaatatt ggacaatggg

4261 ggcaacc

//
